# Lower Limb Kinematics Trajectory Prediction Using Long Short-Term Memory Neural Networks

**DOI:** 10.3389/fbioe.2020.00362

**Published:** 2020-05-08

**Authors:** Abdelrahman Zaroug, Daniel T. H. Lai, Kurt Mudie, Rezaul Begg

**Affiliations:** ^1^Institute for Health and Sport, Victoria University, Melbourne, VIC, Australia; ^2^College of Engineering and Science, Victoria University, Melbourne, VIC, Australia; ^3^Defence Science and Technology Group, Melbourne, VIC, Australia

**Keywords:** LSTM, neural networks, machine learning, forecasting, gait, walking

## Abstract

This study determined whether the kinematics of lower limb trajectories during walking could be extrapolated using long short-term memory (LSTM) neural networks. It was hypothesised that LSTM auto encoders could reliably forecast multiple time-step trajectories of the lower limb kinematics, specifically linear acceleration (LA) and angular velocity (AV). Using 3D motion capture, lower limb position–time coordinates were sampled (100 Hz) from six male participants (age 22 ± 2 years, height 1.77 ± 0.02 m, body mass 82 ± 4 kg) who walked for 10 min at 5 km/h on a 0% gradient motor-driven treadmill. These data were fed into an LSTM model with a sliding window of four kinematic variables with 25 samples or time steps: LA and AV for thigh and shank. The LSTM was tested to forecast five samples (i.e., time steps) of the four kinematic input variables. To attain generalisation, the model was trained on a dataset of 2,665 strides from five participants and evaluated on a test set of 1 stride from a sixth participant. The LSTM model learned the lower limb kinematic trajectories using the training samples and tested for generalisation across participants. The forecasting horizon suggested higher model reliability in predicting earlier future trajectories. The mean absolute error (MAE) was evaluated on each variable across the single tested stride, and for the five-sample forecast, it obtained 0.047 m/s^2^ thigh LA, 0.047 m/s^2^ shank LA, 0.028 deg/s thigh AV and 0.024 deg/s shank AV. All predicted trajectories were highly correlated with the measured trajectories, with correlation coefficients greater than 0.98. The motion prediction model may have a wide range of applications, such as mitigating the risk of falls or balance loss and improving the human–machine interface for wearable assistive devices.

## Introduction

An increasingly useful application of machine learning (ML) is in predicting features of human actions. If it can be shown that algorithm inputs related to actual movement mechanics can predict a limb or limb segment’s future trajectory, a range of apparently intractable problems in movement science could be solved. One such problem is how to anticipate movement characteristics that can predict the risk of tripping, slipping or balance loss. Previous work has investigated balance control using wearable sensors to estimate the body’s centre of mass (CoM) trajectory (Fuschillo et al., 2012). The Internet of things (IoT) has also created a new paradigm of algorithms and systems to predict and subsequently apply interventions to prevent falls (Rubenstein, 2006; Tao and Yun, 2017; Nait Aicha et al., 2018). Perhaps the most valuable motion-prediction application is in the design and control of wearable assistive devices, such as prostheses, bionics and exoskeletons, in which smart algorithms can ensure safer, more efficient integration of the assistive device with the user’s natural limb and body motion (Lee et al., 2017; Rupal et al., 2017).

Previous computational methods have investigated motion trajectory prediction, using position-time inputs and their derivatives (velocity and acceleration). Lower limb trajectory prediction has been implemented in rehabilitation robotics (Duschau-Wicke et al., 2009). Using inverse dynamics, Wang et al. (2011) designed a model for foot trajectory generation using a predefined pelvic trajectory and line fitting 10 data points from a single gait cycle. Also using inverse dynamics, Ren et al. (2007) predicted all segment motions and ground reaction forces from the average forward velocity gait, double stance duration and gait cycle period. Another technique was implemented in the Lower Extremity Powered Exoskeleton (LOPES) device to emulate the trajectories from a healthy limb to the impaired limb (Vallery et al., 2008). Prediction of the lower limb joint angles future trajectory that effectively leads to foot events timing was also investigated in the works of Aertbeliën and De Schutter (2014) and Tanghe et al. (2019) using probabilistic principal component analysis (PPCA).

Recent methods implemented ML algorithms such as artificial neural networks (ANNs) to identify subject gait trajectories to recognise neurological as well as pathological gait patterns (Alaqtash et al., 2011; Horst et al., 2019). Artificial neural networks were also used to improve user intention detection in wearable assistive devices (Jung et al., 2015; Islam and Hsiao-Wecksler, 2016; Moon et al., 2019; Trigili et al., 2019). A variation of ANNs called generalised regression neural networks (GRNNs) was found to be capable of predicting lower limb joint angles (hip, knee and ankle) from the linear acceleration (LA) and angular velocity (AV) of foot and shank segments (Findlow et al., 2008), or from subject gait and anthropomorphic parameters (Luu et al., 2014). Recurrent neural networks (RNNs) and convolutional neural networks (CNNs), which are classes of ANNs, were able to classify human motions and activities (Murad and Pyun, 2017; Han et al., 2019).

Long short-term memory (LSTM) neural networks are a subclass of RNNs, and they have proven success in modelling a wide range of sequence problems, including human activity recognition (Ordóñez and Roggen, 2016), gait diagnosis (Zhao et al., 2018), falls prediction (Nait Aicha et al., 2018) and gait event detection (Kidziński et al., 2019). Long short-term memory autoencoder is an architecture of LSTM that has been implemented in an array of applications such as language translation (Ding et al., 2018) and in forecasting of video frames (Srivastava et al., 2015), weather (Gangopadhyay et al., 2018; Reddy et al., 2018; Poornima and Pushpalatha, 2019), traffic flow (Park et al., 2018; Wei et al., 2019) and stock prices (Li et al., 2018).

Given the potential of lower limb trajectory prediction, no previous work was found that utilised ML techniques to predict future lower limb trajectories using simulated inertial measurement data, which could have a profound impact on human movement science. Simulated measurement data such as the kinematics output from inertial measurement units (IMUs; i.e., LA and AV) offer the opportunity to transcend a predictive model outside the laboratory settings. The aim of this work was to determine whether the kinematics of lower limb trajectories during walking could be reliably extrapolated using LSTM autoencoder neural networks. It was hypothesised that an LSTM autoencoder could reliably forecast multiple time-step trajectories of the lower limb kinematics.

## Materials and Methods

### Collection Protocol

Ethics approval was granted by the Department of Defence and Veterans’ Affairs Human Research Ethics Committee and Victoria University Human Research Ethics Committee (Protocol 852-17). All participants signed a consent form and volunteered freely to participate. Walking data were obtained from six male participants (22 ± 2 years old, 1.77 ± 0.02 m in height, 82 ± 4 kg in mass) who walked for 10 min at 5 km/h on a 0% gradient treadmill. A set of 25 retroreflective markers were attached to each participant in the form of clusters (Findlow et al., 2008). Each cluster comprised a group of individual markers that represent a single body segment (e.g., shank). That included left and right foot (three markers), left shank (four markers), right shank (five markers), left thigh (three markers), right thigh (four markers) and pelvis (three markers). The 3D position of each cluster was tracked using a 14-camera motion analysis system (Vicon Bonita, Version 2.8.2) at 150 Hz. Virtual markers were also established to calibrate the position and orientation of the lower body skeletal system (Garofolini, 2019). Three-dimensional ground reaction force and moment data were collected from a force-plate instrumented treadmill (Advanced Mechanical Technology, Inc., Watertown, MA, United States) at 1,500 Hz.

### Dataset Processing

**FIGURE 1:**
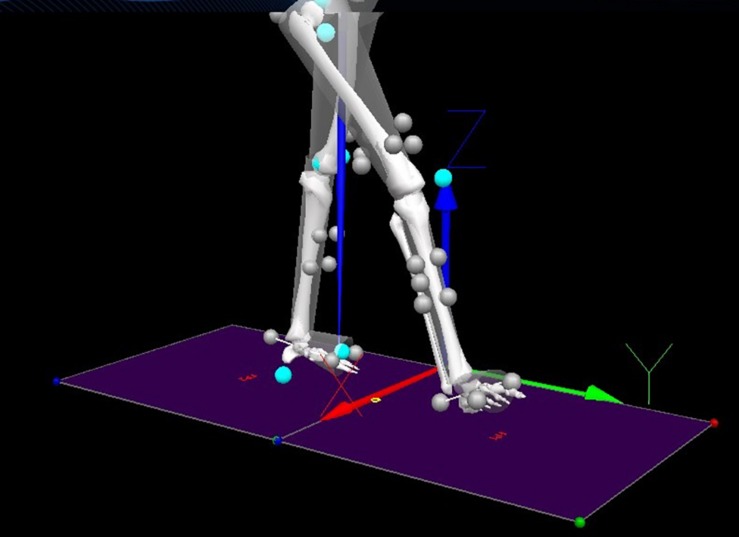
Components (*x*,*y*,*z*) definition and markers setup. Grey balls are retroreflective markers. Turquoise balls are virtual markers.

Recorded 3D positional and force data were processed using Visual 3D (C-motion, Inc, Version 6) to obtain LA and AV. In Visual 3D (Figure 1), the data were firstly filtered using a low-pass digital filter with a 15-Hz cut-off frequency and normalised to mean 0 and standard deviation 1 using standard scores (z-scores), preserving the original data properties. Secondly, raw AV was obtained as the derivative of Euler/Cardan angles (C-motion, 2015), and the raw LA was generated by the double derivative of segment linear displacement using built-in pipeline commands (Hibbeler, 2007). These data (LA and AV) simulated the kinematic outputs from body-mounted IMUs widely used in wearable assistive devices, monitoring lower limb kinematics (Santhiranayagam et al., 2011; Lai et al., 2012), controlling powered actuators (Lee et al., 2017) and recognising human actions (Van Laerhoven and Cakmakci, 2000; Jimenez-Fabian and Verlinden, 2012; Koller et al., 2016).

**FIGURE 2:**
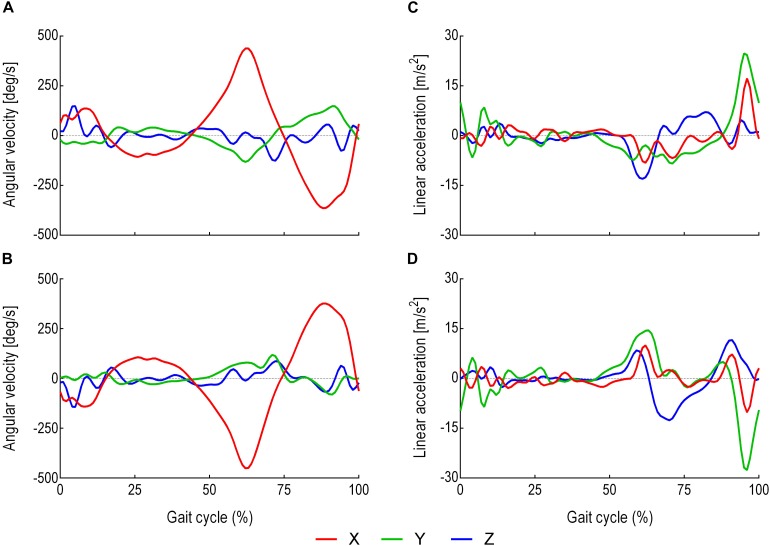
Average thigh and shank LA and AV within a stride. A stride was defined as the interval between two successive heel strikes of the same foot (De Lisa, 1998). **(A)** Thigh three-dimensional AV (direction of the rotation around the *X*-axis). **(B)** Shank three-dimensional AV (direction of the rotation around the *X*-axis). **(C)** Thigh three-dimensional LA (direction of the progression along the *Y*-axis). **(D)** Shank three-dimensional LA (direction of the progression along the *Y*-axis). Red is the *X*-axis. Green is the *Y*-axis. Blue is the *Z*-axis.

As shown in Figure 1, the main direction of movements included the translation along the *Y*-axis (i.e., LA) and the rotation along the *X*-axis (i.e., AV), which were used for LSTM prediction, resulting in four predictor variables: (i) ***Y***_1_ thigh LA, (ii) ***Y***_2_ shank LA, (iii) ***X***_3_ thigh AV and (iv) ***X***_4_ shank AV. The thigh segment was defined as the reference frame to the shank, and the shank segment was defined as the reference frame to the thigh (Figure 2).

### Dataset Description

The data were divided into training and testing sets. The training set comprised 2,665 strides from five participants that included four kinematic feature variables (***Y***_1_, ***Y***_2_, ***X***_3_, ***X***_4_) (N-columns) and 453,060 samples or time steps (M-rows) for each variable. To attain generalisation, a testing set was used that comprised of a single stride from the sixth participant with the four feature variables and 170 samples for each variable.

### Time Series Transformation to a Supervised Learning Problem

**FIGURE 3:**
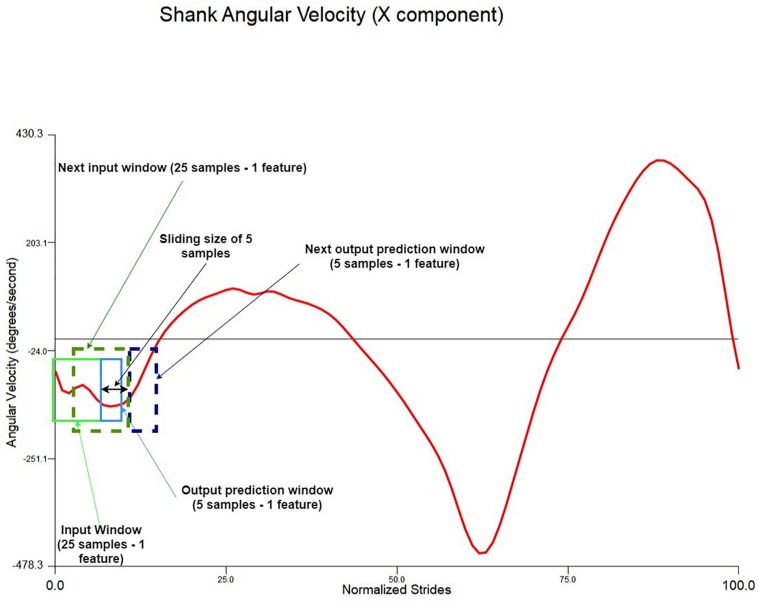
Sliding window illustration example using the normalised shank angular velocity *X*-axis component (one feature). The window in this model is 25 samples and four features and the prediction outputs are five samples of four features.

The inputs to the LSTM were four parallel feature variables and the outputs were the successive four parallel feature variables. Prior to feeding into the LSTM model, the *MxN* training and testing datasets were transformed to a 3D dataset using a sliding window technique (Banos et al., 2014). The sliding window comprised of an input window, an output window and a sliding size. The input window consists of *M* samples and *N* features, so as the output window. The input window is the input data to the LSTM model, and the output window is the future prediction output from the LSTM model. The sliding size is how much of *M* samples that both the input and the output windows are sliding forward with (see Figure 3). The sliding size (*M* samples) was always equal to the output size.

### Recurrent Neural Networks

**FIGURE 4:**
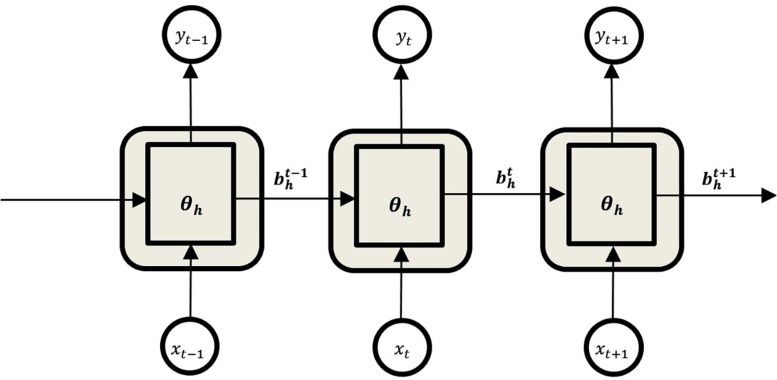
Unfolded structure of the Recurrent Neural Network.

While multiple layer perceptrons (MLPs) consider all inputs as independent, RNNs are designed to work with time series data (Ordóñez and Roggen, 2016). RNNs are a class of ANN architecture designed specifically to model sequence problems and exploit the temporal correlations between input data samples (Elman, 1990; Murad and Pyun, 2017). It contains feedback connections between each of its units, which enables the network to relate all the previous inputs to its outputs (Figure 4).

The forward pass equations from the inputs to the outputs of the RNN are given as follows.

For the hidden units:

(1) $$a_{h}^{t}=\sum_{i=1}^{I}w_{ih}x_{i}^{t}+\sum_{h^{\prime }=1}^{H}w_{h^{\prime }h}b_{h^{\prime }}^{t-1}$$

and differentiable activation functions are then applied:

(2) $$b_{h}^{t}=\theta_{h}\left(a_{h}^{t}\right)$$

The network input to output units:

(3) $$a_{k}^{t}=\sum_{h=1}^{H}w_{hk}b_{h}^{t}$$

where

$a_{h}^{t}$ is the sum of inputs to unit *h* at time *t*, $b_{h}^{t}$ is the activation of unit *h* at time *t*, θ_*h*_ is the non-linear and differentiable activation function of unit *h*, $a_{k}^{t}$ is the sum of all inputs to output unit *k* at time *t*, $x_{i}^{t}$ is the input *i* at time *t*, *w*_*ih*_ is the connection weights between input unit *i* and hidden unit *h*, *w*_*h′ h*_ is the connection weights between the previous hidden state *h*′ and itself *h* and *w*_*hk*_ is the connection weights between the hidden state *h* and the output unit *k*. Bias was neglected for simplicity.

### LSTM Networks

**FIGURE 5:**
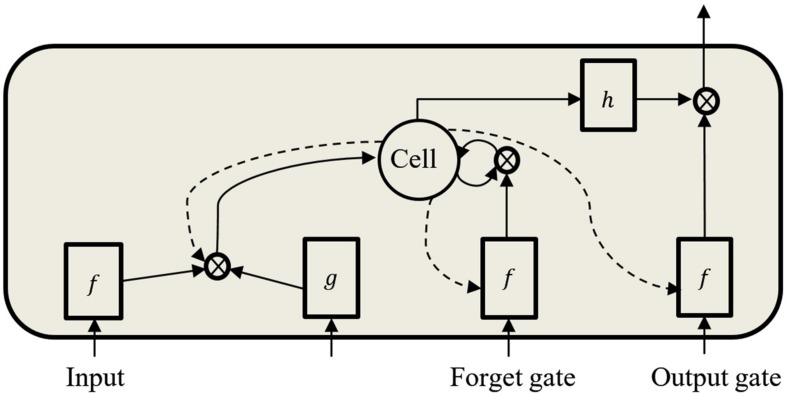
Standard LSTM memory cell with peephole connections.

As the input data propagates through the standard RNN’s hidden connections to the output units, it either slowly attenuates or amplifies exponentially, referred to, respectively, as vanishing or exploding gradients (Bengio et al., 1994; Hochreiter et al., 2001). The problems with this approach are that the vanishing gradient prevents the network from learning long-term dependencies and the exploding gradient leads to weights oscillation. These difficulties have been addressed using gradient norm clipping to tackle the exploding gradient and a soft constraint to deal with the vanishing gradient (Pascanu et al., 2013). The LSTM design addresses these problems by maintaining a memory cell *C* (Figure 5) that enables the network to retain information over a longer period by using an explicit gating mechanism (Hochreiter and Schmidhuber, 1997; Graves, 2012; Karpathy et al., 2015).

Each LSTM cell has an input gate, forget gate, and output gate. The input gate dictates the information used to update the memory state, and the forget gate decides which information to discard or remove from the cell. The final gate specifies the information to output based on the cell input and memory. All gates are designed such that information is exchanged from inside and outside the block (Figure 5). Furthermore, each memory block contains three peephole-weighted connections (dotted lines in Figure 5), which are the input weight *w*_*cι*_, the output weight *w*_*cω*_ and the memory state *w*_*cϕ*_. The functions *f*, *g* and *h* are usually tanh or logistic sigmoid activation functions (Graves, 2012). Below are the network equations (Graves, 2012) that govern the LSTM architecture used:

Input gates:

(4) $$a_{\iota }^{t}=\sum_{i=1}^{I}w_{i\iota }x_{i}^{t}+\sum_{h=1}^{H}w_{h\iota }b_{h}^{t-1}+\sum_{c=1}^{C}w_{c\iota }s_{c}^{t-1}$$

(5) $$b_{\iota }^{t}=f\left(a_{\iota }^{t}\right)$$

Forget gates:

(6) $$a_{\phi }^{t}=\sum_{i=1}^{I}w_{i\phi }x_{i}^{t}+\sum_{h=1}^{H}w_{h\phi }b_{h}^{t-1}+\sum_{c=1}^{C}w_{c\phi }s_{c}^{t-1}$$

(7) $$b_{\phi }^{t}=f\left(a_{\phi }^{t}\right)$$

Cells:

(8) $$a_{c}^{t}=\sum_{i=1}^{I}w_{ic}x_{i}^{t}+\sum_{h=1}^{H}w_{hc}b_{h}^{t-1}$$

(9) $$s_{c}^{t}=b_{\phi }^{t}s_{c}^{t-1}+b_{\iota }^{t}g\left(a_{c}^{t}\right)$$

Output gates:

(10) $$a_{c}^{t}=\sum_{i=1}^{I}w_{iw}x_{i}^{t}+\sum_{h=1}^{H}w_{hw}b_{h}^{t-1}+\sum_{c=1}^{C}w_{cw}s_{c}^{t}$$

(11) $$b_{w}^{t}=f\left(a_{w}^{t}\right)$$

Cell outputs:

(12) $$b_{c}^{t}=b_{w}^{t}h\left(s_{c}^{t}\right)$$

where *w*_*ij*_ is the weight of the connection from unit *i* to unit *j*; $a_{j}^{t}$ is the network input to unit *j* at time *t*; $b_{j}^{t}$ is the activation of unit *j* at time *t*; ι, ϕ, ω respectively stand for the input gate, the forget gate and the output gate; *C* is the memory cell; *w*_*cι*_, *w*_*cϕ*_, *w*_*cω*_ are peephole weights; $s_{c}^{t}$ is the state of cell *C* at time *t*; *f* is the input, output and forget gates activation function; *g* and *h* are the cell input and output activations, respectively; *I* is the number of inputs; *H* is the number of cells in the hidden layer; and index *h* is the cell outputs from other blocks in the hidden layer. Bias was neglected for simplicity.

### Design of the LSTM Model

**FIGURE 6:**
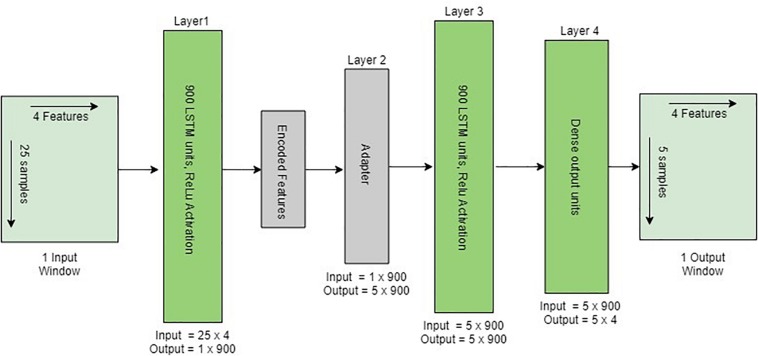
Structure of the implemented encoder–decoder LSTM architecture given one input window. The adapter converts the 2D encoded features into 3D output to be adopted by LSTM. The last layer is a fully connected dense layer for outputting one window prediction.

The implemented model was based on the autoencoder LSTM, a neural network architecture composed of an encoder and a decoder (Ding et al., 2018). The encoder encodes the input variable length vector into a fixed length feature vector that captures the attributes of the variable length vector. The LSTM decoder decodes the encoded fixed length feature vector back into a variable length vector (Figure 6). The final layer is a fully connected dense (feedforward) mechanism for outputting predictions. The network weights and biases were updated at the end of each batch using an adaptive moment estimation (Adam) optimisation algorithm (Kingma and Ba, 2014) with mean absolute error (MAE) as an optimisation criterion. A single batch consists of 100 input/output windows. The activation for all LSTM layers was set to a rectified rectilinear unit (ReLU) activation function (Nair and Hinton, 2010). The LSTM autoencoder model was implemented in Google Colab as well as Amazon Web Services (AWS) using Python 3 (Libraries: Keras, Numpy, Pandas and Scikit learn).

### Evaluation Metrics

To evaluate the network quality, three parameters were considered to calculate how closely the network predicted variable trajectories $\hat{y_{j}}$ (***Y***_1_, ***Y***_2_, ***X***_3_, ***X***_4_) were to the actual variable trajectories *y*_*j*_ (***Y***_1_, ***Y***_2_, ***X***_3_, ***X***_4_) across the *n* samples:

1. MAE given as:
  (13) $$MAE=\frac{1}{n}\sum_{j=1}^{n}\left|y_{j}-\hat{y_{j}}\right|$$
2. Mean squared error (MSE) given as:
  (14) $$MSE=\frac{1}{n}\sum_{j=1}^{n}{\left(y_{j}-\hat{y_{j}}\right)}^{2}$$
3. Correlation coefficient (CC) given as:
  (15) $$P=\frac{cov\left(y,\hat{y}\right)}{std\left(y\right)\times std\left(\hat{y}\right)}$$
  where *std*() is the standard deviation and $cov\left(y,\hat{y}\right)$ is the covariance between variables *y* and $\hat{y}$.

## Results

Using the sparse grid search approach, the model’s hyperparameters were tuned to determine the optimum model design (least MAE), including the number of epochs, batch size, layers and cells. The optimum model was then trained for 50 epochs (repetitions), and performance evaluated on the test set using MAE, MSE and the CC. The test set was a single stride that consisted of 170 samples. Initial 25 samples were used from the preceding cycle in order to start predicting the trajectories of the single stride.

### Model Performance With Different Input Window Sizes

**FIGURE 7:**
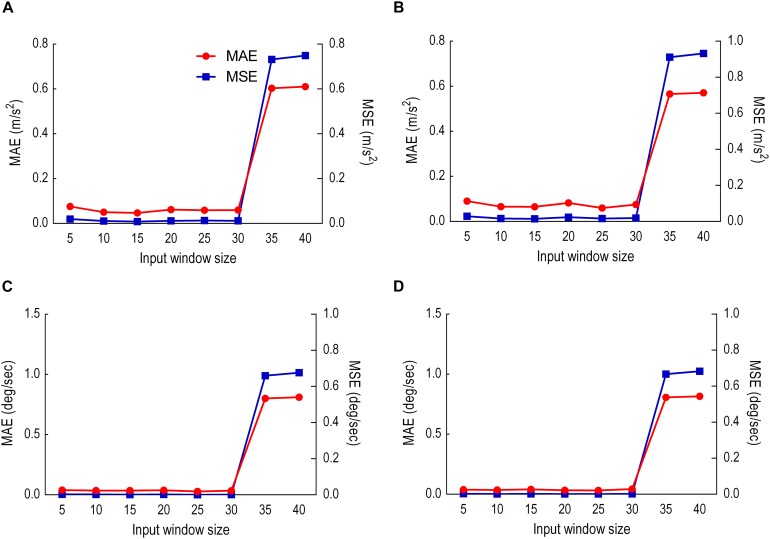
Model performance with different input window sizes. Red is MAE. Blue is MSE. **(A)** Thigh LA (***Y***_1_). **(B)** Shank LA (***Y***_2_). **(C)** Thigh AV (***X***_3_). **(D)** Shank AV (***X***_4_).

The size of the input window was varied eight times at five sample intervals (5–40 samples) to demonstrate the optimum input window size (least error). The output sliding window was fixed to five samples prediction. The model performance is shown in Figure 7 where the impact of each input window size on the prediction of each variable is computed.

### Model Performance With Five Samples Prediction

**FIGURE 8:**
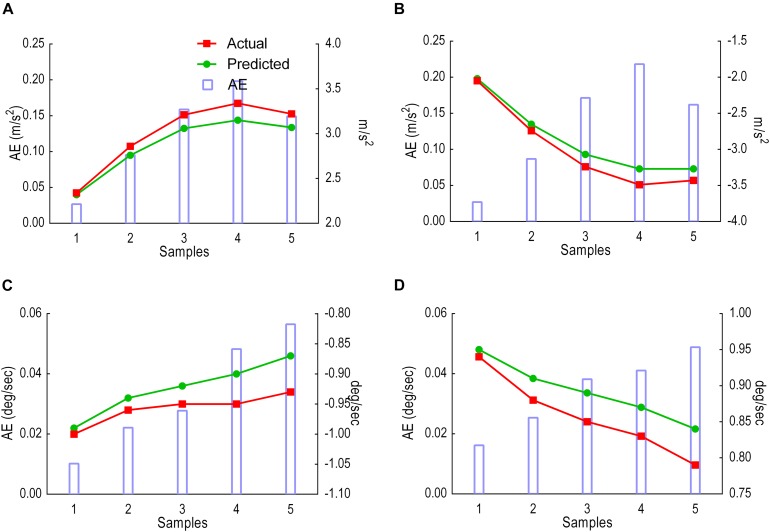
Model performance for the first window, showing predicted trajectories (green) and actual trajectories (red). Columns represent the absolute error (AE) for the five predicted samples. **(A)** Thigh LA ***Y***_1_. **(B)** Shank LA ***Y***_2_. **(C)** Thigh AV ***X***_3_. **(D)** Shank AV ***X***_4_

**FIGURE 9:**
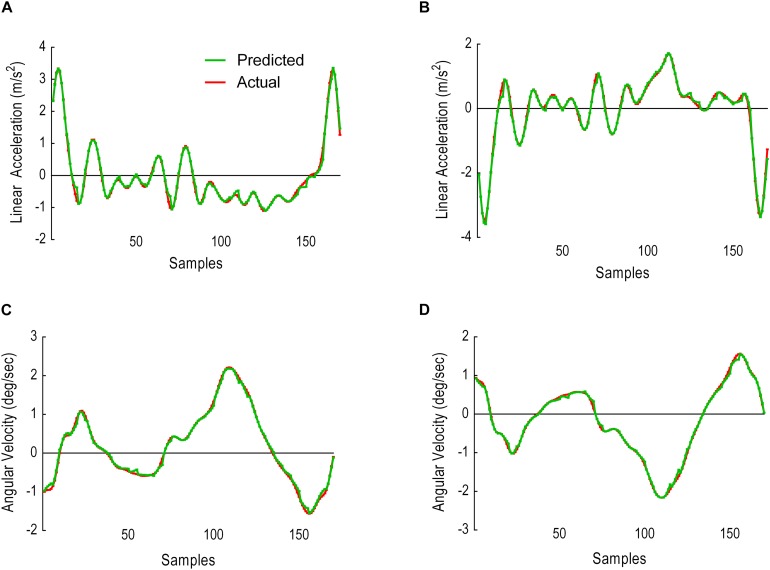
Model performance over the entire gait cycle when five samples prediction window is used. The figure shows predicted trajectories (orange) and actual trajectories (blue). **(A)** Thigh LA ***Y***_1_. **(B)** Shank LA ***Y***_2_. **(C)** Thigh AV ***X***_3_. **(D)** Shank AV ***X***_4_.

This sliding window comprised of 25 samples input and 5 samples prediction output. Results were given in two analyses: (i) predicted versus actual trajectories including the absolute error (AE) for each sample in the first output window (Figure 8) and for the whole gait cycle (Figure 9) and (ii) performance metrics (MAE, MSE and CC) for the first window of five samples (Table 1) and for all windows combined (Table 2).

**TABLE 1:** Model performance for predicting the first five stride samples.

Feature	MAE	MSE	CC
***Y*_1_**	0.125 m/s^2^	0.019 m/s^2^	0.99
***Y*_2_**	0.133 m/s^2^	0.022 m/s^2^	0.99
***X*_3_**	0.032 deg/s	0.001 deg/s	0.98
***X*_4_**	0.033 deg/s	0.001 deg/s	0.99

**TABLE 2:** Model performance for predicting the complete stride using an input window size of 25 samples and an output window size of 5 samples.

Feature	MAE	MSE	CC
***Y*_1_**	0.047 m/s^2^	0.006 m/s^2^	0.99
***Y*_2_**	0.047 m/s^2^	0.006 m/s^2^	0.99
***X*_3_**	0.028 deg/s	0.001 deg/s	0.99
***X*_4_**	0.024 deg/s	0.001deg/s	0.99

### Model Performance With 10 Samples Prediction

**FIGURE 10:**
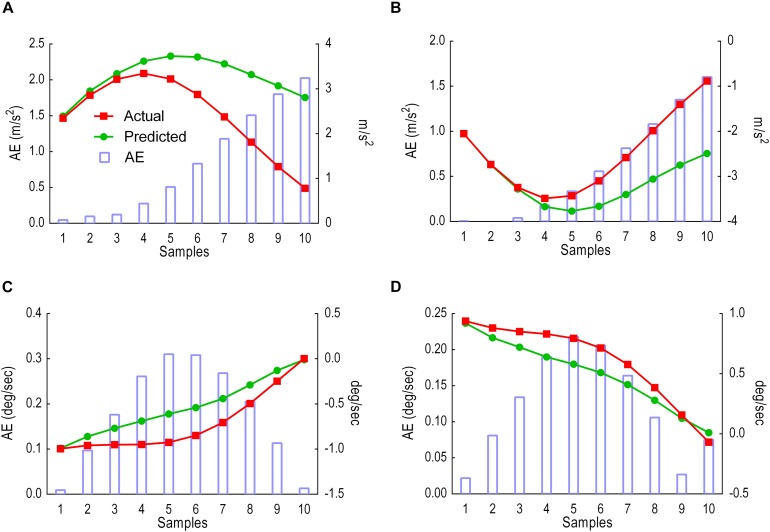
Model performance for the first window, showing predicted trajectories (green) and actual trajectories (red). Columns represent the AE for the 10 predicted samples. **(A)** Thigh LA ***Y***_1_. **(B)** Shank LA ***Y***_2_. **(C)** Thigh AV ***X***_3_. **(D)** Shank AV ***X***_4_.

**FIGURE 11:**
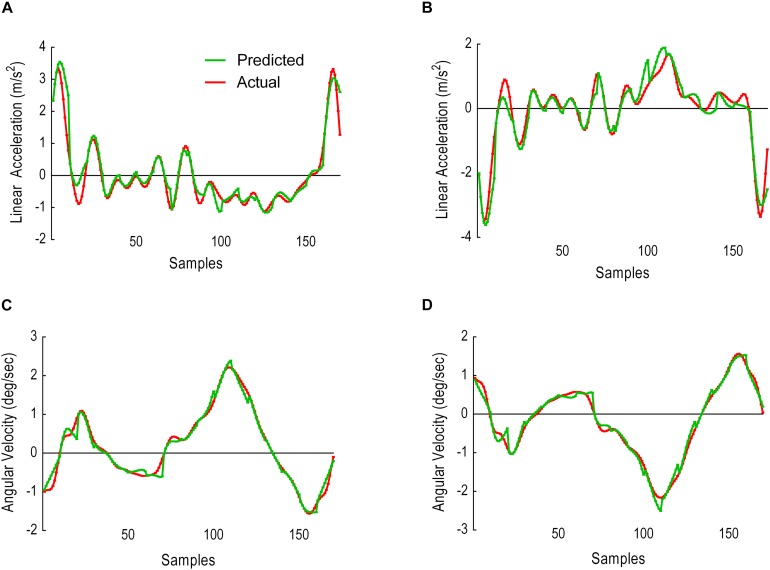
Model performance over the entire gait cycle when 10 samples prediction window is used. The figure shows predicted trajectories (orange) and actual trajectories (blue). **(A)** Thigh LA ***Y***_1_. **(B)** Shank LA ***Y***_2_. **(C)** Thigh AV ***X***_3_. **(D)** Shank AV ***X***_4_.

This sliding window comprised of 25 samples input, 10 samples prediction output. Figure 10 illustrates the results as predicted versus the actual trajectories including the AE for each sample in the first output window, whereas Figure 11 displays the results for the whole gait cycle. Performance metrics (MAE, MSE and CC) for the first window of 10 samples are presented in Table 3 and for all windows combined in Table 4.

**TABLE 3:** Model performance for predicting the first 10 stride samples.

Feature	MAE	MSE	CC
***Y*_1_**	0.839 m/s^2^	1.206 m/s^2^	0.52
***Y*_2_**	0.596 m/s^2^	0.667 m/s^2^	0.75
***X*_3_**	0.176 deg/s	0.042 deg/s	0.94
***X*_4_**	0.122 deg/s	0.019 deg/s	0.96

**TABLE 4:** Model performance for predicting the complete stride using an input window size of 25 samples and an output window size of 10 samples.

Feature	MAE	MSE	CC
***Y*_1_**	0.170 m/s^2^	0.096 m/s^2^	0.96
***Y*_2_**	0.202 m/s^2^	0.096 m/s^2^	0.96
***X*_3_**	0.079 deg/s	0.015 deg/s	0.98
***X*_4_**	0.086 deg/s	0.014 deg/s	0.98

## Discussion

Our aim was to develop and evaluate an LSTM autoencoder model to predict the trajectories of four kinematic variables (***Y***_1_, ***Y***_2_, ***X***_3_, ***X***_4_), simulating the output from wearable sensors (IMU). The predicted kinematic feature variables, LA and AV, for the shank and thigh were reliably predicted up to 10 samples or time steps, i.e., up to 60 ms in the future. A 60-ms prediction of future trajectories adds a feedforward term to an assistive device controller rather than being reactive and predominantly relying on feedback terms (i.e., sensory information; Tanghe et al., 2019). This enables the assistive device to adapt to changes in human gait, allowing smoother synchronization with user intentions and minimising interruptions when the user changes their movement pattern (Elliott et al., 2014; Zhang et al., 2017; Ding et al., 2018; Zaroug et al., 2019). A known future trajectory might also monitor the risk of balance loss, tripping and falling, in which impending incidents can be remotely reported for early intervention (Begg and Kamruzzaman, 2006; Begg et al., 2007; Nait Aicha et al., 2018; Hemmatpour et al., 2019; Naghavi et al., 2019). Since 60 ms falls in the range of slow (60–120 ms) and fast (10–50 ms) twitch motor units (Winter, 2009), this would enable wearable devices such as IMUs to alert (e.g., by audio/visual signal) an elderly user about an imminent risk of tripping and potentially gives them a chance to adjust their gait accordingly.

In contrast to the 1- to 2-s window for human activity recognition proposed by Banos et al. (2014), no window has previously been suggested for forecasting human movement trajectories (Banos et al., 2014). In addressing this limitation, the present project input and output sliding windows were tested to discover the optimum prediction model. The input window was varied from 5 to 40 samples, whereas the output window was fixed at 5 samples during each test. Results showed that both MAE and MSE increased after 25 samples for all variables except for the thigh LA *Y*_1_ in which 15 samples scored lowest. Due to the majority score, 25 samples were fixed, and the output window size manipulated between 5 and 10 samples. Prediction error MAE and MSE gradually increased across the first 5 and 10 sample prediction windows, indicating better prediction early in the stride cycle. This prediction horizon suggests that an output window exceeding five samples may not be sufficiently reliable for forecasting gait trajectories. LA-predicted trajectories began to deviate earlier than AV, possibly due to the double derivative generating a noisier signal.

Across the stride cycle, an output window of 5 samples showed better model performance (lower MAE scores) than the 10-sample output window, particularly when there is less noise in the predicted signal for all variables. Predictions of five samples for all variables achieved high CC (0.99) and maintained below MAE 0.048 deg/s and 0.029 m/s^2^. These result parameters are different from those of earlier work (Findlow et al., 2008; Luu et al., 2014). The difference is in the type of predicted data (lower limb joint angles of the hips, knees and ankles) and in the type of output, which was not a forecast, but rather a prediction of joint angles from the LA and AV of the lower limb segments. Nonetheless, the work presented in this paper showed higher CC values than the earlier works (Findlow et al., 2008; Luu et al., 2014) at the intersubject test. Overall, the LSTM model was able to learn the trajectories and generalise across participants. This generalisation is invaluable to adapt algorithm performance to a wider population in assistive devices, particularly when each user responds differently to the same device (Zhang et al., 2017).

This study was limited to the walking movement with a 60-ms prediction horizon and healthy participants walking at 5 km/h. The speed was imposed to report the feasibility of whether lower limb future trajectories are predictable. In future work, the model would be developed to accommodate a higher gait variance from more participants and other populations, such as female, older adults and individuals with gait disorders walking at their preferred as well as slower and faster speeds (Winter, 1991). More participants (i.e., stride examples) would potentially improve the model performance to predict trajectories above 60 ms and also provide a more comprehensive validation set, a strategy to find the optimum number of epochs and avoid model overfitting (Graves, 2013). The LSTM autoencoder can be made flexible by automating the input/output window size depending on the detected human activity, which revamps the LSTM capacity to recognise a wider range of human action transitions, such as slow to fast walking. Although LSTM autoencoders described here were able to learn and predict future data points, further research is needed to explore other LSTM architectures, such as bi-directional LSTM (Graves and Schmidhuber, 2005). Bi-directional LSTM can be useful in forward and backward modelling of sequential data, giving further insights into sequential pattern modelling (Liu and Guo, 2019; Zhang et al., 2019).

## Conclusion

This study confirmed the possibility of predicting the future trajectories of human lower limb kinematics during steady-state walking, i.e., thigh AV, shank AV, thigh LA and shank LA. An input window of 25 samples and an output window of 5 samples were found to be the optimum sliding window sizes for future trajectories prediction in LSTM. The LSTM model prediction horizon was better able to forecast the earlier sample trajectories and was also able to learn trajectories across different participants. Further work is required to systematically investigate the effects of tuning the model’s hyperparameters, including layers and cells, optimisation algorithms and learning rate. Future work could focus on automating input/output window size and using predicted kinematics to identify discrete gait cycle events such as heel strike and toe-off (Kidziński et al., 2019). Long short-term memory methods for human movement prediction have applications to balance loss, falls prevention and controlling of assistive devices.

## Data Availability Statement

The datasets generated for this study are available on request to the corresponding author.

## Ethics Statement

The studies involving human participants were reviewed and approved by Associate Professor Deborah Zion Chair of Victoria University Human Research Ethics Committee. The patients/participants provided their written informed consent to participate in this study.

## Author Contributions

AZ wrote the manuscript and coded the ML model. AZ, DL and RB contributed to research and ML model design and analysis. KM and RB designed the biomechanics experiment. KM and AZ collected and analysed the biomechanics data. All authors provided critical feedback on the manuscript and read and approved the final manuscript.

## Conflict of Interest

The authors declare that the research was conducted in the absence of any commercial or financial relationships that could be construed as a potential conflict of interest.
